# The current status and history of AFLAS

**DOI:** 10.1186/s42826-022-00144-1

**Published:** 2022-11-24

**Authors:** Noriyuki Kasai

**Affiliations:** Professor Emeritus, Tohoku University, Sendai, Miyagi 9808575 Japan

**Keywords:** AFLAS congress, Council meeting, Laboratory animal science, Education and training system

## Abstract

The Asian federation of laboratory animal science associations (AFLAS) was established on November 29, 2003, and will celebrate its 20th anniversary in 2023. During this time, the number of AFLAS member associations and societies increased from six founders to eleven, and eight AFLAS congresses and 19 council meetings were held. In addition, the education and training system of laboratory animal science and technology funding program to support the activities of AFLAS member associations or societies started in 2015. Unfortunately, the COVID-19 pandemic had a great impact on the activities of AFLAS, and the 10th Congress which was scheduled to be held in Thailand in 2021 had to be canceled. AFLAS must have its members work together to overcome this difficult situation and further develop.

## Background

The Asian Federation of Laboratory Animal Science Associations (AFLAS) was established on November 29, 2003 and will celebrate its 20th anniversary in 2023. During this time, AFLAS now has eleven associations and societies from the six founders of associations and societies of laboratory animal science in Asia and has held eight biennial congresses and 19 annual council meetings, although the 9th Congress was unfortunately canceled due to the COVID-19 pandemic (Table 1).

**Table 1:** Chronological table of AFLAS congress and council meeting

Year	President	AFLAS Congress, Venue & Date	Council Meeting, Venue & Date
2003	Dr. Shigeru Sugano(JALAS)		Gakushi Kaikan in Tokyo, Japan, on November 29, 2003
2004	**”**	1st Congress at Nagasaki Brick Hall in Nagasaki, Japan from May 21–22, 2004	Nagasaki Brick Hall in Nagasaki, Japan on May 21, 2004
2005	Dr. Yong-Soon Lee(KALAS)		Seoul University in Seoul, Korea on June 3, 2005
2006	**”**	2nd Congress at International Convention Center Jeju in Jeju, Korea from August 30 to September 1, 2006	International Convention Center Jeju in Jeju, Korea on August 31, 2006
2007	Dr. Qin Chuan (CALAS)		Grand Epoch City in Beijing, China on August 27, 2007
2008	**”**	3rd Congress at Grand Epoch City in Beijing, China from September 27–29, 2008	Grand Epoch City in Beijing, China on September 29, 2008
2009	Dr. Chou C. Hong (CSLAS)		Taipei International Convention Center in Taipei, Taiwan on September 11, 2009
2010	**”**	4th Congress at Taipei International Convention Center, Taipei, Taiwan from November 9–11, 2010	Taipei International Convention Center in Taipei, Taiwan on September 9, 2010
2011	Dr. Parntep Ratanakorn (TALAS)		Bangkok International Trade & Exhibition Centre in Bangkok, Thailand on September 16, 2011
2012	**”**	5th Congress at Bangkok International Trade & Exhibition Centre in Bangkok, Thailand from October 10–12, 2012	Bangkok International Trade & Exhibition Centre in Bangkok, Thailand on September 11, 2012
2013	Dr. Abdul Rahim Mutalib (LASAM)		The Royale Chulan Hotel in Kuala Lumpur, Malaysia on November 9, 2013
2014	**”**	6th Congress at the Royale Chulan Hotel in Kuala Lumpur, Malaysia from November 11–12, 2014	The Royale Chulan Hotel in Kuala Lumpur, Malaysia on November 11, 2014
2015	Mr. Yi-Quan Tay (SALAS)		The Raffles City Convention Centre in Singapore on November 28, 2015
2016	**”**	7th Congress at the Raffles City Convention Centre in Singapore from November 8–10, 2016	The Raffles City Convention Centre in Singapore on November 9, 2016
2017	Dr. Arvind Ingle (LASA India)		The International Center for Genetic Engineering and Biotechnology in New Delhi, India on November 24, 2017
2018	**”**	8th Congress at Hotel ITC Gardenia in Bangalore, India from November 28 to December 1, 2018	Hotel ITC Gardenia in Bangalore, India on Nobember 29, 2018
2019	Dr. Parntep Ratanakorn (TALAS)		The Empress Hotel in Chiang Mai, Thailand on June 20, 2019
2020	**”**	9th Congress was postponed to the next year due to the COVID-19 pandemic	The meeting was held by e-mail from August to September 2020 due to the COVID-19 pandemic
2021	**”**	9th Congress was canceled due to the COVID-19 pandemic	The meeting was held online using Zoom on November 19, 2021, due to the COVID-19 pandemic
2022	Dr. KilSoo Kim (KALAS)		

As described in AFLAS statutes, the aims are to promote through international cooperation Asian congresses on laboratory animal science for the purpose of reviewing the scientific, technical and educational problems in laboratory animal science, to develop other relevant activities in the interest of laboratory animal science and to contribute to animal welfare. AFLAS has served these aims well thus far, and has contributed to the development of the laboratory animal science in Asia.

AFLAS records can be found on its website [1]. In particular, the detailed minutes of the council meetings can be found on the minutes page of this site.

## The foundation of AFLAS

AFLAS was established on November 29, 2003 by six associations and societies of laboratory animal science on a national or regional basis in Asia. However, it took a long time to reach the goal of establishment.

### The international exchange meeting in Japan in May 2001

The Japanese Association for Laboratory Animal Science (JALAS, the president was Dr Shigeru Sugano) and the Japanese Congress of Laboratory Animal Science and Technology 2001 (JCLAST2001, the president was Dr. Kazuyoshi Maejima) organized a symposium entitled "Laboratory Animal Science in Asian Countries -Present and Future" in the congress held at Yokohama, Japan on May 9, 2001. The aim of this symposium was to exchange information and to develop good relationship between the associations of laboratory animal science in Asian countries or regions. The delegates from six associations and societies of laboratory animal science from China, Japan, Korea, the Philippines, Taiwan and Thailand participated in the symposium. After the symposium, they discussed about establishing AFLAS.

All the delegates agreed to the proposal of a new federation of Asian laboratory animal science associations/societies to be organized to improve the quality of laboratory animal science in Asia. In addition, it was discussed that scientists and academic journals should be exchanged among the member associations/societies of the new federation, and a new journal of laboratory animal science should be published. Finally, it was decided that each of delegates discusses the foundation of the Federation of Asian Laboratory Animal Science Associations (FALASA) (tentative name) at their respective associations/societies, and that the plan for establishing a new federation should be announced to other countries that were absent from JCLAST2001, e.g., India, Bangladesh, Indonesia, Malaysia, etc.

### The international award by the Asian fund of JALAS

The international award by the Asian fund of JALAS played an important role in uniting the associations and societies of laboratory animal science in Asia. The Asian fund was founded as a memorial of the 50th anniversary of the founding of JALAS on May 11, 2001. JALAS formally asked Dr. Sang-Seop Han, the President of the Korean Association for Laboratory Animal Science (KALAS) to recommend a KALAS award-winning candidate at the ceremony of the anniversary on December 1, 2001. The first award ceremony was held at the 49th annual meeting of JALAS in Nagoya on May 23, 2002, and five young scientists from five countries and regions in Asia were given the international JALAS award.

### Process of the founding of the new federation

JALAS President Sugano and Dr. Kasai, the chair of the JALAS international communication committee, visited Seoul, Korea on October 5, 2002 and discussed the establishment of the new federation with KALAS President Sang-Seop Han and the board members of KALAS. The two sides agreed to establish a new federation.

JALAS made a plan to have a meeting to found the new federation on March 2003 and sent a draft of the founding regulation, such as a statute and a bylaw (ver. 1), to the associations and societies that are scheduled to join on January 7, 2003. However, both the meeting for the new federation and the second award ceremony of the international award of JALAS were postponed because of the SARS epidemic in Asia.

### Foundation of the new federation, AFLAS, and the first council meeting for AFLAS in November 2003 in Tokyo, Japan

Finally, the first Council meeting for the establishment of the new federation in Asia was held at the Gakushi Kaikan annex on the Hongo campus of the University of Tokyo, Tokyo, Japan on November 29, 2003. Five associations or societies, namely, KALAS (Korea), the Philippine Association for Laboratory Animal Science (PALAS), the Chinese Taipei Society of Laboratory Animal Science (CSLAS, Taiwan), the Thai Association for Laboratory Animal Science (TALAS) and JALAS (Japan), gathered in Tokyo, and discussed the establishment of the new federation of laboratory animal science in Asia. It was unanimously decided and named the “Asian Federation of Laboratory Animal Science Associations” and abbreviated as “AFLAS”.

The five associations or societies became founding members. Unfortunately, the representative of the Chinese Association for Laboratory Animal Science (CALAS) could not attend the meeting due to visa application trouble, but all members agreed that CALAS was also a founding member.

The STATUTE and BYLAWS of AFLAS were also established, and the first executive board members were elected as follows: President: Dr. Shigeru Sugano (JALAS); Vice-Presidents: Dr. Yong-Soon Lee (KALAS); Secretary General: Dr. Noriyuki Kasai (JALAS); and Auditor: Dr. Chin-En Tsai (CSLAS). In addition, it was decided that the First AFLAS Congress would be held in Nagasaki, Japan on May 22, 2004, and the Second Congress of AFLAS would be planned in Korea in 2006 and prepared by KALAS as the next organizer.

## AFLAS congresses and council meetings

According to the AFLAS statutes, AFLAS shall arrange for the Asian Congress on Laboratory Animal Science to be held once every two years. In addition, AFLAS holds a council meeting every year with the participation of the council members of every association and society of AFLAS. I introduce all of these congresses and council meetings that have been held to date.

### The First Congress and the 2004 Council meeting of AFLAS in Japan

The First Congress and the 2004 Council meeting of AFLAS were held in Nagasaki, Japan, from May 21–22, 2004 under the first AFLAS President Shigeru Sugano with the 51st annual meeting of JALAS and its president, Dr. Hiroshi Sato.

Two symposia were held in the congress as follows:

Symposium I “Quality Control of Laboratory Animals: Current Status in Asian Countries”; and Symposium II “Technicians of Experimental Animals in Asia”.

The delegates of five associations, CALAS, KALAS, JALAS, PALAS and TALAS, were represented at the council meeting on May 21, 2004. AFLAS President Shigeru Sugano (JALAS) presided over the meeting. The amount of the annual membership fee from the 2005 fiscal year was decided. Dr. Yong-Soon Lee (KALAS) was elected as the second president for 2004–2006. The members confirmed that the next council meeting of AFLAS would be held in Seoul, Korea in June 2005 and that the Second Congress of AFLAS would be held in Korea in 2006.

### The 2005 council meeting of AFLAS in Korea

The council meeting was held at Seoul University in Seoul, Korea, on June 3, 2005. Only three members attended, namely, CALAS, KALAS and JALAS. AFLAS President Dr. Yong-Soon Lee presided over the meetings of the council. At the meeting, basic matters of meeting management, such as annual membership dues and how to use the budget, were discussed. In addition, AFLAS President Prof. Yong-Soon Lee and KALAS President Dr. Yang-Seok Oh explained the preparation of the congress to be held in Jeju Island, Korea in 2006.

### The Second Congress and the 2006 council meeting of AFLAS in Korea

The Second Congress and the 2006 Council meeting of AFLAS were held at the International Convention Center Jeju in Jeju, Korea from August 30 to September 1, 2006 under AFLAS President Prof. Yong-Soon Lee. It was organized by KALAS, with the President and Chair Dr.Yang-Seok Oh. Approximately 500 people were gathered from all over the world, and ten symposia, one workshop and 78 posters were presented at the congress. Two symposia were organized by the International Council for Laboratory Animal Science (ICLAS) and AAALAC International.

The delegates of five AFLAS member associations and societies were represented at the council meeting held on August 31, 2006. In addition, two representatives from the Laboratory Animal Science Association of Malaysia (LASAM) and the Singapore Association for Laboratory Animal Science (SALAS) attended as observers. The amount of the annual membership fee from the 2006 fiscal year was decided. Dr. Qin Chuan (CALAS) was elected as the third president from 2006 to 2008. The members confirmed that the next council meeting of AFLAS would be held in Beijing, China in October 2007 and that the Third Congress of AFLAS would be held in Beijing in 2008. The meeting also decided that the Fourth Congress of AFLAS would be held in Taipei in 2010.

### The 2007 council meeting of AFLAS in China

A meeting was held on August 27, 2007 in Grand Epoch City, Beijing, China. The delegates of four AFLAS member associations and societies, specifically, CALAS, CSLAS, JALAS and KALAS were represented at the meeting. AFLAS President Dr. Qin Chuan presided over the meeting. The president introduced the preparation of the 3rd AFLAS Congress, and the date and venue were decided as September 27–29, 2008 at Citic Guoan Grand Epoch City in Beijing.

A new website for the AFLAS office was decided to be created and maintained by Secretary General Dr. N. Kasai for the continuous information distribution of AFLAS.

### The Third Congress and the 2008 council meeting of AFLAS in China

The Third Congress and the 2008 council meeting of AFLAS were held at Grand Epoch City, Beijing, China from September 27–29, 2008 under AFLAS President Dr. Qin Chuan with the 8th Annual Meeting of CALAS. Nine general reports, 6 presentations for the Asian Laboratory Animal Science Forum, and 8 oral sessions, including 388 papers and 63 posters, were presented at the congress.

The delegates of six AFLAS associations and societies were represented at the council meeting held on September 29, 2008. In addition, three representatives from LASAM, SALAS and the Laboratory Animal Scientists' Association (India) (LASA India) attended as observers. At the meeting, we discussed joining three associations to AFALS and voted. All the member associations or societies agreed for them to join AFLAS. AFLAS thus consisted of nine associations or societies in Asia.

KALAS donated the relief fund of 1,500,000 Korean Won (approximately 9500 RMB) for the people made destitute by the earthquake disaster in Sichuan Province, China, and CALAS awarded Prof Yong-Soon Lee, the immediate past-president of AFLAS, for his great efforts for the 2nd AFLAS Congress.

For the Fifth AFLAS Congress in 2012, two associations announced their candidacy as host associations at the meeting. This was to be decided at the council meeting in 2009, which would be held in Taipei, Taiwan. Dr. Chou C. Hong (CSLAS) was elected as the fourth AFLAS President from 2008 to 2010.

### The 2009 council meeting of AFLAS in Taiwan

The meeting was held on September 11, 2009, at the Taipei International Convention Center in Taipei, Taiwan. AFLAS President Dr. Chou C. Hong presided over the meeting. The president introduced the preparation of the Fourth AFLAS Congress and the venue.

At the meeting, TALAS was elected as the host association for the 2012 congress. In addition, Secretary General Dr. Noriyuki Kasai announced that the official AFLAS website was created [1].

### The Fourth Congress and the 2010 council meeting of AFLAS in Taiwan

The Fourth Congress and the council meeting of AFLAS were held at the Taipei International Convention Center in Taipei, Taiwan from November 9–11, 2010 under AFLAS President Dr. Chou C. Hong (CSLAS) with the 5th AMMRA Meeting and 11th CSLAS Annual meeting*.* Eighty-three speakers were invited from all over the world. A total 114 oral presentations, including 6 keynote sessions and 167 poster papers were presented in the congress.

All nine AFLAS member associations and societies attended the council meeting held on September 9, 2010. In addition, Dr.Dondin Sajuthi, a representative of a laboratory animal association in Indonesia, also attended as an observer. President Dr. Chou C. Hong presided over the meeting. LASAM (President Dr. Abdul Rahim Mutalib) was elected unanimously as the host association for the 6th AFLAS Congress in 2014. The Indonesian association representative Dr.Dondin Sajuthi expressed his desire to join his association with AFLAS, and AFLAS Secretary General Dr. Noriyuki Kasai was to contact the association continuously.

It was decided for AFLAS to join the BOT of AAALAC International. The executive board members for 2010–2012 were elected and Dr. Parntep Ratanakorn (TALAS) was designated as the AFLAS President.

### The 2011 council meeting of AFLAS in Thailand

The meeting was held on September 16, 2011, at the Bangkok International Trade & Exhibition Centre (BITEC) in Bangkok, Thailand*.* Six AFLAS member associations and societies, specifically, CSLAS, JALAS, KALAS, LASAM, PALAS and TALAS, attended the meeting. In addition, Dr. Joko Pamungkas (the Indonesia Society for Laboratory Animal Science, IALAS) also attended as an observer. AFLAS President Dr. Parntep Ratanakorn presided over the meeting. The president introduced the preparation of the Fifth AFLAS Congress and the venue.

The admission of IALAS to AFLAS was discussed. Since IALAS was not yet legally registered by the Indonesia authority, AFLAS decided to put on hold their application until its formal registration. As soon as the formal registration is completed, IALAS will become a member of AFLAS.

### The Fifth Congress and the 2012 council meeting of AFLAS in Thailand

The Fifth Congress and the 2012 council meeting of AFLAS were held at BITEC in Bangkok, Thailand from October 10–12, 2012 under AFLAS President Dr. Parntep Ratanakorn (TALAS).

The main objective of the AFLAS congress in 2012 was to bring the global standard of laboratory animal science and management to Asian laboratory animal science personnel by organizing state-of-the-art continuing educational programs that can be applied to laboratory animal facilities in all regions. The highlights of the main program were the keynote lecture and 15 sessions of 38 stellar special topic lectures and panel discussions. The program brought 35 experts from the U.S.A., Europe and Asia. Approximately 100 interesting abstracts were submitted for platform and poster scientific paper presentations related to laboratory animal science, new animal models of human diseases, and other studies or activities conducted in Asia and other regions.

The council meeting was held on October 11, 2012*,* at BITEC in Bangkok, Thailand*.* AFLAS President Dr. Parntep Ratanakorn presided over the meeting. All nine association or society members attended the meeting in addition to IALAS as an observer. As the Indonesian authority formally granted the legal document of IALAS in April 2012, the AFLAS council members unanimously approved and accepted the formal membership of IALAS. With the inclusion of IALAS in the association, AFLAS had ten member associations and societies.

The election of a host association or society for the 7th AFLAS Congress in 2016 was held and decided that SALAS was to be the host. The election of the council and executive board members for 2013–2014 was held, and Dr. Abdul Rahim Mutalib (LASAM) was elected as AFLAS President. AFLAS decided to support Mongolia and Vietnam to develop laboratory animal science there and establish their laboratory animal acience Associations.

### The 2013 council meeting of AFLAS in Malaysia

The meeting was held on November 9, 2013*,* at The Royale Chulan Hotel in Kuala Lumpur, Malaysia. AFLAS President Dr. Abdul Rahim Mutalib (LASAM) presided over the meeting. Nine out of the ten AFLAS member associations and societies attended the meeting. The president introduced the preparation of the Sixth AFLAS Congress and the venue.

The suggestion of hosting and organizing an irregular AFLAS symposium between the AFLAS congresses was accepted. The purpose was to strengthen the relationship among AFLAS members and to develop the field of laboratory animal science in Asia as a whole by having more opportunities for academic meetings at flexible conferences.

It was also decided that AFLAS would join ICLAS as an affiliate member in response to ICLAS’s invitation.

CALAS proposed establishing an education and training system for laboratory animal technology at AFLAS. The members asked CALAS to propose a concrete idea for a way to establish such an education and training system.

### The Sixth Congress and the 2014 council meeting of AFLAS in Malaysia

The Sixth Congress and the 2014 council meeting of AFLAS were held at the Royale Chulan Hotel in Kuala Lumpur, Malaysia from November 11–12, 2014 under AFLAS President Dr. Abdul Rahim Mutalib (LASAM).

The goal of the congress was centered on addressing issues in animal welfare and animal biology, capacity development and education in laboratory animal science. Cutting-edge topics in anticancer research, drug development, aging and translational medicine, imaging modalities, and emergency response and mitigation strategies for animal facilities were also the focus of AFLAS 2014, all of which were presented by 44 internationally renowned speakers.

The 2014 council meeting was held on November 11, 2014*,* at the same venue. AFLAS President Dr. Abdul Rahim Mutalib (LASAM) presided over the meeting. All ten AFLAS member associations and societies attended the meeting.

LASA India was elected as the host association or society for the 8th AFLAS Congress in 2018. The holding of “2015 AFLAS–KALAS Joint Symposium” as an AFLAS irregular meeting and the support of 2000USD by AFLAS were decided.

The election of the council and executive board members for 2015–2016 was held, and Mr. Yi-Quan Tay (President of SALAS) was elected as AFLAS President.

Three expected associations from Mongolia, Vietnam and Sri Lanka were introduced to be AFLAS members. Only the Sri Lanka association was established at this time. The Sri Lanka Association for Laboratory Animal Science (SLALAS) was established in December 2012, and AFLAS received a letter from President Prof. Mangala Gunatilake indicating its intent to join AFLAS. The council meeting decided to recommend SLALAS to join AFLAS.

### The education and training system of laboratory animal science and technology (ETALAST) funding program

According to the decision at the council meeting in Kuala Lumpur, Malaysia　in 2013, we discussed concrete measures for ETLAST at the 2014 council meeting. In addition, the committee of ETALAST was established to create the proper funding system and select appropriate programs.

The aim of the program is to support activities for the training and education of laboratory animal science, including techniques, animal welfare and experimental ethics, in the AFLAS member associations or societies. This program started at the AFLAS council meeting held in Singapore in 2015.

The program supports the travel expenses of lecturer(s) or expert(s) for training and education activities of each association or society of AFLAS. By 2019, 15 applicants were funded.

### The 2015 council meeting of AFLAS in Singapore

The meeting was held on November 28, 2015*,* at the Raffles City Convention Centre (RCCC) in Singapore. AFLAS President Mr. Yi-Quan Tay (SALAS) presided over the meeting. Seven out of the ten member associations or societies and one observer, SLALAS, attended the meeting. The president introduced the preparation of the Seventh AFLAS Congress and the venue.

The admission of SLALAS to AFLAS was discussed. After Dr. Mangala Gunatilake, SLALAS Founding President and Managing Director of SLALAS introduced her new association, the council members discussed and unanimously approved SLALAS to join AFLAS. SLALAS became the eleventh member association of AFLAS. Welcome and congratulations!

The ETALAST funding program started, and the AFLAS office began to accept application forms from AFLAS member associations and societies for programs from December 2015 to November 2016.

Dr. Qin Chuan (CALAS) proposed an urgent subject for discussion: the establishment of an AFLAS journal. We decided to discuss this issue in the future.

### The Seventh Congress and the 2016 council meeting of AFLAS in Singapore

The Seventh Congress and the 2016 council meeting of AFLAS were held at RCCC in Singapore from November 8–10, 2016 under AFLAS President Mr. Yi-Quan Tay (SALAS). The sessions covered topics such as the 3Rs (which form the cornerstones of laboratory animal science), infectious disease, pathology, biomedical imaging, and different animal models, including rodents, swine and aquatics, all of which were presented by 47 internationally renowned speakers. In addition, a pre-congress from November 6–7 and a post-congress from November 11–13 were held for interesting topics.

The 2016 council meeting was held on November 9, 2016 at the same venue. AFLAS President Yi Quan Tay (SALAS) presided over the meeting. All eleven member associations and societies attended the meeting.

The first ETALAST funding program was carried out, and three programs from SLALAS, LASA India and KALAS were selected.

The election of the council and executive board members for 2017–2018 was held, and LASA India President Dr. Arvind Ingle was elected as AFLAS President. TALAS was unanimously elected as the host association or society for the 9th AFLAS Congress in 2020.

### The 2017 council meeting of AFLAS in India

The meeting was held on November 24, 2017 at the International Center for Genetic Engineering and Biotechnology in New Delhi, India. AFLAS President Dr. Arvind Ingle (LASA India) presided over the meeting. Nine out of eleven member associations and societies attended the meeting. Dr. SG Ramachandra, Organizing Secretary of the AFLA Congress 2018 and the former vice-president of AFLAS, presented the preparation of the 8th AFLAS Congress and the venue.

The second ETALAST funding program was carried out, and three applicants were selected from the SLALAS, CALAS and PALAS programs.

### The Eighth Congress and the 2018 council meeting of AFLAS in India

The congress was held at Hotel ITC Gardenia in Bangalore, India from November 28 to December 1, 2018 under AFLAS President Dr. Arvind Ingle (LASA India).

The congress consisted of a three-day main meeting and a one-day pre-congress workshop by AAALAC International, and a cross-functional sharing of information, a better understanding of the requirements of the regulatory authorities, and welfare and laboratory animal professionals were expected to be highlighted and discussed. The participants gathered from all over the world, and 58 symposia and 52 papers were presented orally and in posters at the congress.

The 2018 council meeting was also held at Hotel ITC Gardenia in Bangalore, India on November 29, 2018, and all AFLAS members attended. The third ETALAST funding program was carried out, and three applicants were selected from the CALAS and two LASAM programs.

KALAS was elected as the host association or society for the 10th AFLAS Congress in 2022. In addition, AFLAS President Dr. Parntep Ratanakorn (TALAS) and the executive board members for 2019–2020 were elected.

### Discussion about the AFLAS journal

The idea of publishing an AFLAS journal was discussed at the 2004 council meeting, shortly after AFLAS was founded. In addition, at the 2006 council meeting, Dr. Qin Chuan (CALAS) explained that financial support from Chinese authorities was possible. Since 2015, this issue has been actively taken up and discussed at council meetings. Dr. Qin Chuan made a concrete proposal for the publication of an AFLAS journal, and it was decided to continue discussions about it at future council meetings. In 2016, based on the preliminary discussions between CALAS and JALAS, a working committee (Chair: Professor Je Kyung Seong, KALAS) was set up to discuss this issue intensively at the council meeting. Then, on October 10, 2018, members of the working committee gathered and discussed this issue at Shan・gri-la Hotel Qingdao in Qingdao, China.

The discussion thus far has been that five academic journals have been published by AFLAS member academic societies (JALAS, KALAS, CALAS, LASA India and PALAS), three of which are published in English as follows: “Experimental Animals” published by JALAS; “Laboratory Animal Research” published by KALAS; and the “Journal of Laboratory Animal Science” published by LASA India. In addition, a new academic journal, “Animal Models and Experimental Medicine” was published in English in March, 2018, by CALAS.

Finally, it was decided that the AFLAS journal should be a group of English journals published by AFLAS member associations or societies, and that any associations that have their own journal can apply for collaboration with AFLAS and be an AFLAS journal. The term used for the collaboration between AFLAS and the journals is “in cooperation with AFLAS”.　Other requirements for a collaboration with AFLAS are listed in the minutes of the 2018 council meeting.

### The 2019 council meeting of AFLAS in Thailand

The meeting was held on June 20, 2019, at the Empress Hotel in Chiang Mai, Thailand*.* AFLAS President Dr. Parntep Ratanakorn (TALAS) presided over the meeting. Six out of eleven member associations and societies attended the meeting. Dr. Montip Gettayacamin introduced the preparation of the Ninth AFLAS Congress from June 22–26, 2020 at the venue of the Empress Hotel in Chiang Mai, Thailand.

The fourth ETALAST funding program was carried out, and three applicants were selected from the IALAS, LASAM and PALAS programs.

### The 2020 council meeting of AFLAS by e-mail

The meeting was held by e-mail from August to September 2020 due to the COVID-19 pandemic. The Ninth AFLAS Congress scheduled for June 22–26, 2020 was postponed to June 21–27, 2021 due to the COVID-19 pandemic. The term of the current executive board member was extended until the end of the Ninth AFLAS Congress due to postponement of the AFLAS congress. The period of the ETALAST funding program in 2020 was also extended from the end of March 2021 to March 2022.

It has been decided that the Tenth AFLAS Congress in Korea will be rescheduled for September 13–15, 2023 and that the eleventh and subsequent congresses will be held every two years from 2025 onward, such as 2025, 2027, and 2029, etc.

### The 2021 council meeting of AFLAS by zoom

The meeting was held online using Zoom on November 19, 2021, due to the COVID-19 pandemic. All eleven member associations and societies participated, and AFLAS President Dr. Parntep Ratanakorn (TALAS) presided over the meeting.

Unfortunately, AFLAS President Parntep Ratanakorn declared that the Ninth AFLAS Congress that was planned to be held in Chaing Mai, Thailand in June 2021 would be canceled because the COVID-19 pandemic had not ended in the world.

KALAS President Dr. KilSoo Kim, Dr. Seung-Hyun Oh and Dr. Je Kyung Seong introduced the preparation status of the Tenth AFLAS Congress in 2023 in Korea. The date is September 2023, and the venue is Jeju Island, Korea.

It was decided in a previous council meeting that the ETALAST funding program in 2021 be carried out as planned. However, the COVID-19 pandemic had not improved even in 2021. In addition, the AFLAS office did not receive any reports of carrying out the 2020 programs from any associations. Therefore, the period of the ETALAST funding program in 2020 was extended again to March 2023. It was decided that the ETALAST funding program in 2021 be cancelled. However, the ETALAST funding program in 2022 will be carried out as planned.

The election of the council and executive board members for 2021–2023 was carried out, and Dr. KilSoo Kim (KALAS) was elected as AFLAS President. Secretary General Dr. Noriyuki Kasai announced his intention to resign his position at AFLAS, and it was decided to consider this issue at a new executive board at a later date and consult with members at the council meeting. It was also decided that a new secretary general would be examined by the new executive board at a later date, and the results would be discussed at the council meeting.

## Conclusions

It has been approximately 20 years since AFLAS was founded in 2003. During this time, AFLAS has increased its membership from 6 founding associations and societies to 11 and has held eight biennial congresses and 19 annual council meetings. Unfortunately, due to the COVID-19 pandemic, the 9th Congress scheduled to be held in Thailand in 2021 was cancelled. However, KALAS will hold the 10th AFLAS Congress in 2023. KALAS held the 2nd AFLAS Congress in Jeju in 2006. The president was Prof. Yong-Soon Lee of Seoul National University, and 500 people from Europe, Korea and other part of Asia participated. At this time, the situation is quite difficult because of the COVID-19 pandemic, but I expect that many people from Korea and around the world will participate in the AFLAS congress.

Finally, I would like to state the directions that AFLAS should take for future development. First of all, it is necessary to participate in Asian countries with laboratory animal science association such as Vietnam and Mongolia. Second, AFLAS is currently a member of international societies such as the International Council for Laboratory Animal Science (ICLAS) and the Association for Assessment and Accreditation of Laboratory Animal Care International (AAALAC International). Moreover, we should seek more active interaction with regional federations such as the Federation of European Laboratory Animal Science Associations (FELASA), the American Association for Laboratory Animal Science (AALAS), and the Australian and New Zealand Laboratory Animal Association (ANZLAA). Third, we need to establish some working committees to promote animal welfare and education of care and use of laboratory animals in Asia and extend the range of AFLAS activities. Fourth, in order to guarantee the above activities, it is necessary to review the membership fee, increase income, and achieve stable financial management. I hope that AFLAS member associations and societies will make efforts to develop laboratory animal science in Asia with reference to these.

It is a personal matter, but I retired as the Secretary General and Vice-President at the council meeting in November 2021. I had been Secretary General since the founding of AFLAS in 2003 and Vice President since 2008 to support the development of AFLAS. During this time, I helped hold eight AFLAS Congresses and 19 council meetings.

I would like to thank all of the presidents and members of the AFLAS member associations and societies for being able to continue and develop this society. I hope that this paper contributes to the future development of AFLAS.

## Data Availability

Not applicable.

## Abbreviations

AFLAS: The Asian Federation of laboratory animal science associations
ETALAST: The education and training system of laboratory animal science and technology
JALAS: The Japanese association for laboratory animal science
JCLAST2001: The Japanese congress of laboratory animal science and technology 2001
FALASA: The Federation of Asian laboratory animal science associations
KALAS: The Korean association for laboratory animal science
PALAS: The Philippine association for laboratory animal science
CSLAS: The Chinese Taipei society of laboratory animal science
TALAS: The Thai association for laboratory animal science
CALAS: The Chinese association for laboratory animal science
ICLAS: The international council for laboratory animal science
LASAM: The laboratory animal science association of Malaysia
SALAS: The Singapore association for laboratory animal science
RCCC: The Raffles city convention centre
LASA India: The laboratory animal scientists’ association (India)
IALAS: The Indonesia society for laboratory animal science
SLALAS: The Sri Lanka association for laboratory animal science
